# Oviposition Preferences and Behavior of Wild-Caught and Laboratory-Reared Coconut Rhinoceros Beetle, *Oryctes rhinoceros* (Coleoptera: Scarabaeidae), in Relation to Substrate Particle Size

**DOI:** 10.3390/insects9040141

**Published:** 2018-10-15

**Authors:** Megan Manley, Michael J. Melzer, Helen Spafford

**Affiliations:** Department of Plant and Environmental Protection Sciences, University of Hawaiʻi at Mānoa, Honolulu, HI 96822, USA; manleym@hawaii.edu (M.M.); melzer@hawaii.edu (M.J.M.)

**Keywords:** oviposition, Hawaiʻi, invasive species, *Oryctes rhinoceros*

## Abstract

The coconut rhinoceros beetle (CRB), *Oryctes rhinoceros* (L.) (Coleoptera: Scarabaeidae), has become one of the most important coconut and oil palm pests. This species was detected attacking coconut palms on Oʻahu, Hawaiʻi in December 2013, and an eradication program was initiated. One of the major challenges for eradication has been the identification of new breeding sites. Information on the factors influencing oviposition is needed to aid in finding sites likely to host the immature stages of this insect. In this study, a series of choice tests were conducted to assess the oviposition preferences of both laboratory-reared and wild-caught CRB. Mated females, of both lab-reared and wild-caught beetles, were offered for oviposition a choice between sand and two mulch substrates, one with small and one with large particle sizes. Both types of CRB laid eggs preferentially in substrate of small particle size rather than large and none laid eggs in sand. Lab-reared and wild-caught CRB differed in their oviposition behavior and size. These results can be used to aid in the identification of breeding sites for management programs and eradication efforts.

## 1. Introduction

Native to south and southeast Asia [1], the coconut rhinoceros beetle (CRB), *Oryctes rhinoceros*, has been unintentionally introduced throughout the South Pacific to countries and territories such as Papua New Guinea, Samoa, American Samoa, Tonga, Fiji, Wallis Island, Micronesia, the Cocos Islands, Saipan, and Guam. Without control, adult beetles can decimate coconut palm populations and are now considered one of the most important coconut and oil palm pests worldwide [2]. When CRB was detected in Hawaiʻi in December 2013, an eradication program was implemented with a main objective being the identification and removal of breeding sites [3]. However, this proved to be one of the major challenges in mitigating CRB populations. Adult beetles can lay their eggs in various substrates, typically rich in decomposing plant material, found at the base of palm fronds or in piles on the ground, that the larvae then feed upon [1,4,5]. This allows for a wide range of media that could potentially house different life stages of the beetle. Although many aspects of the biology and reproduction of CRB have been studied [4,5,6,7], it is unclear what factors stimulate a female to choose a particular substrate in which to oviposit. Oviposition preference has been previously demonstrated among soil-nesting Scarabaeidae. In scarabs, such as the Japanese beetle and rose chafer, oviposition is influenced by soil moisture as well as texture [8]. In other Coleoptera, particle size is also a factor that influences oviposition. Grain size was a factor influencing oviposition behavior of two species of tiger beetles, with *Cicindela hirticollis* Casey (Coleoptera: Carabidae) species preferring finer sand and *C. oregona* LeConte species preferring coarser sand [9]. Thus, we expect female CRB will exhibit substrate preferences and discern between different particle sizes.

As part of the eradication program in Hawaiʻi, a CRB colony has been established and used to research and test potential management tools. Laboratory rearing is known to impact insect performance, traits, and behavior [10,11,12,13,14,15] and thus is of concern when colony-reared individuals are then used in bioassays and behavioral studies that influence management decisions.

The primary purpose of this study was to determine if CRB displays substrate preferences for oviposition, as this information will be useful to the eradication program in Hawaiʻi and elsewhere where CRB is under active management. As an additional component of this study, we compared the oviposition of lab-reared and wild-caught CRB, as well as their biometrics in relation to fecundity.

## 2. Materials and Methods

### 2.1. Insect Colony

The adult *O*. *rhinoceros* beetles used in this study were either collected from the field using panel and barrel traps [3] and maintained in a colony (wild-caught), or reared under laboratory conditions, from eggs produced by field-collected adults, to adult stage (laboratory-reared). Both groups of adults were maintained separately within a colony initiated by the Hawaiʻi Department of Agriculture in 2015 and later transferred to a containment laboratory at the University of Hawaiʻi at Mānoa campus. The colony had been maintained for two years prior to the start of the experiments.

Wild-caught adults were used to produce the laboratory-reared insects. Two generations of laboratory-reared beetles had been successfully reared at the time that these experiments began. However, only beetles from the F1 generation were used in these experiments. To produce laboratory-reared CRB, groups of five adult males and 10 adult females (1:2) were placed in plastic boxes (41.9 cm L × 33.0 cm W × 16.8 cm H and 43.2 cm L × 28.3 cm W × 6.5 cm H) (Sterilite Corporation, Townshend, MA, USA) with a medium of 50:50 commercial mulch (Menehune Magic Mulch, Hawaiian Earth Products, Waimānalo, HI, USA) and coconut coir (Pacific Growers Supplies, Inc., Waimānalo, HI, USA), and allowed to mate freely and oviposit. Starting in 2017, all medium was switched to mulch acquired from the University of Hawaiʻi at Mānoa Landscaping Department, comprising of trimmings and organic matter. Eggs produced by these matings were dug out by hand-sifting through the medium, and were transferred to plastic containers (41.0 cm L × 28.6 cm W × 27.6 cm H) (Sterilite Corporation, MA, USA) also containing the same mulch. The collected eggs were left to hatch, grow to the third instar, and were then transferred to individual Nalgene containers (ThermoFisher Scientific, Cleveland, OH, USA) that were placed within a biological incubation chamber at a setting of 29 °C with a RH of 69% (Model I-41LLVL, Percival Scientific, Inc., Perry, IA, USA). The larvae periodically received replacement mulch for feeding and were left to pupate until adult emergence. This produced the F1 generation of laboratory-reared beetles used in these experiments.

Male and female laboratory-reared pupae emerged as adults in the individual containers. Thereafter, they were grouped in separate colony boxes according to sex. The boxes were identical to those used for wild-caught beetles. Adult male and female CRB can be distinguished by the presence of a fuzzy patch of hairs on the ventral surface at the tip of the abdomen on the females and a generally larger horn found on the head of males that is less pronounced on the females.

All adult beetles, wild-caught and laboratory-reared, were fed on an artificial diet made of vanilla-flavored whey protein, sugar, agar and water that was melted, mixed together and set as a soft gelatinous cube using ice-cube trays.

### 2.2. Experimental Insects

Adult female beetles were selected from the wild-caught and laboratory-reared groups for use in the experiments. Wild-caught beetles were selected from the colony. Given that these insects had been collected from the field, their age and reproductive history was unknown. The wild-caught beetle colony was established in June 2015. As beetles were caught in the field they were brought to Hawaiʻi Department of Agriculture Plant Quarantine and were introduced and maintained within the colony. These female beetles may have laid eggs in nature prior to collection. Females used in the experiments were selected from colony boxes in which eggs had been laid. It was therefore assumed that these female beetles were mated and fecund. The fecundity of individuals in the colony had not been tracked.

Laboratory-reared adult females were reared from eggs produced by the wild-caught beetles. The laboratory-reared females had a known diet and developmental history when selected. To produce mated laboratory-reared females, adult beetles were removed from female-only colony boxes and placed in a mating arena with a laboratory-reared male for 3 days prior to placement in the choice arena. The mating arena consisted of a (19.4 cm L × 16.5 cm W × 11.4 cm H) plastic container (Sterilite Corporation, MA, USA) containing the 50:50 mulch and coconut coir mixture. Laboratory-reared beetles were on average 42 days post-eclosion.

### 2.3. Substrates for Oviposition

Beetles were given a choice of different substrates for oviposition: sand, and mulches with either large or small particle size. To produce substrates of different particle size, mulch was oven-dried at 120 °C for a minimum of 24 h and mixed thoroughly, then sifted through one of two sizes of steel screen and rehydrated. Steel screen size 1.6 mm was used to produce a substrate with small particles (Phifer Incorporated, Tuscaloosa, AL, USA), and size 12.0 mm was used to produce a substrate with large particles (Everbilt, Atlanta, GA, USA). Substrate with the larger particle size was placed in one compartment of the arena, in another, small particle-sized substrate, and in the third, sand (The QUIKRETE Companies, Inc., Atlanta, GA, USA). All substrates were made to be level within the arena.

### 2.4. Choice Test

A series of choice tests were conducted under laboratory conditions to determine whether female oviposition is influenced by substrate. A single female beetle was placed in an arena and given a choice among two mulches that varied in particle size, and a sand substrate in which to lay her eggs. The female beetle was held in the arena for 7 days and allowed to dig freely throughout the arena. At the end of the 7 days, all three substrates were hand-sifted and the eggs were removed. The site and number of eggs laid in each of three available substrates was recorded at the end of 7 days. Only the total number of eggs laid during the 7-day period was recorded. To minimize disturbance for the beetles there were no per/day collections. Each female was scored as laying all eggs in either substrate (large or small particle size, or sand), some eggs in multiple substrates within the same trial, or no eggs in any substrate.

The arena for the choice tests consisted of a wooden chamber with a lockable lid (50.8 cm L × 30.5 cm W × 33.0 cm H) with three inner compartments, one containing sand (25.4 cm L × 17.3 cm W × 25.7 cm H) and two containing different mulch choices (25.4 cm L × 13.3 cm W × 25.7 cm H). Plastic containers (25.4 cm L × 12.7 cm W × 27.3 cm H) (InterDesign, Salon, OH, USA) were placed inside the oviposition choice compartments for better ease of removing the substrate and examining for eggs. Basic mulch used for the experiments was gathered from the University of Hawaiʻi at Mānoa Landscaping Department. Each choice arena was kept with the lid closed within a biological incubation chamber at a setting of 29 °C with a RH of 69% (Model I-41LLVL, Percival Scientific, Inc., IA). Three replicates were placed in the incubation chamber at the same time. At the end of the period, the containers were emptied and refilled with new substrate prior to the introduction of a new female.

A total of 24 laboratory-reared and 24 wild-caught females were tested (*n* = 48).

### 2.5. Biometrics

All 24 wild-caught beetles were measured for what was defined in this study as body length (mm), width (mm), depth (mm), and weight (g). The same biometric data were collected from 18 laboratory-reared beetles. Using a caliper, beetle body length was measured from the anterior tip to the posterior tip, width was measured on the widest part of the abdomen, depth was measured from the area of the body with the greatest girth, and weight was measured using a digital balance (A&D Weighing, San Jose, CA, USA).

### 2.6. Data Analysis

To address the question as to whether there is a preference for large particle versus small particle substrate, the oviposition pattern for each beetle (wild-caught and laboratory-reared) was scored and analyzed using categorical data analysis techniques. A single female beetle was scored as either laying her eggs in one mulch substrate or the other, sand, multiple substrates in the same trial, or none. The preferences of wild-caught and laboratory-reared beetle oviposition substrates were compared using a Chi-Squared Test of Independence. A Chi-Squared Goodness of Fit Test was used for wild-caught and laboratory-reared beetles separately to assess if oviposition would be equally distributed across substrates. Within each beetle type, the number of eggs laid per beetle in each substrate was compared using a nonparametric Wilcoxon Ranked-Sum Test.

We also sought to evaluate the biological difference between the two groups of beetles. The total number of eggs laid by each beetle type was compared using a one-way ANOVA. The biometrics of the two groups of beetles was compared using a one-way MANOVA followed by one-way ANOVA tests for each parameter: length, width, depth and weight. Female beetle biometrics were related to fecundity using a backwards-elimination stepwise multiple regression to assess if each biometric parameter could be used as a predictor of fecundity. All statistical analyses were conducted using JMP Pro 13 (SAS Institute Inc., Cary, NC, USA).

## 3. Results

### 3.1. Choice Test

**Figure 1:**
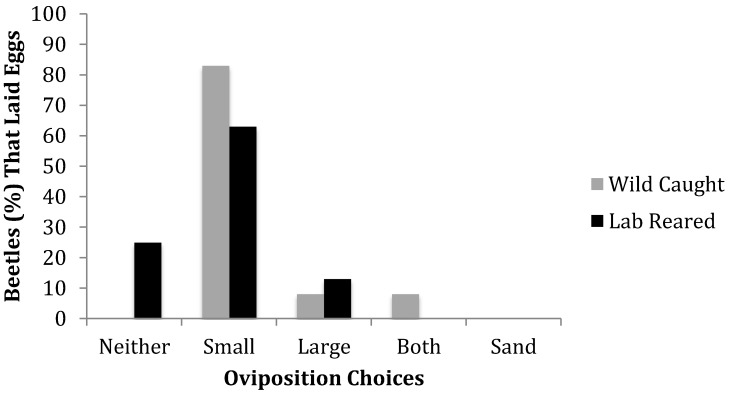
Percent of CRB laying no eggs (neither), laying all their eggs in either mulch substrate (small or large), within both mulch substrates at once (both), or in sand. *n* = 24 wild-caught; *n* = 24 lab-reared.

Generally, CRB beetles preferred to lay their eggs in mulch with small particle size (Figure 1, Table 1). Regardless of developmental history, the majority of eggs were laid in small particle size mulch: 73% of all beetles laid all of their eggs in small particle size mulch and 94% of all eggs laid were in small particle size mulch. No beetles laid any eggs in sand.

However, laboratory-reared and wild-caught beetles exhibited different oviposition patterns when given a choice between large and small particle size mulches and sand (Figure 1; χ^2^ = 8.91, df = 3, *p* = 0.0305). Laboratory-reared beetles were the only beetle type to exhibit the behavior of laying 0 eggs within a 7-day observation period (Figure 1). Whereas, all wild-caught beetles laid eggs. The laboratory-reared beetles that laid eggs only laid eggs in a single-substrate i.e., one of the mulches. Whereas, some wild-caught beetles laid eggs in both mulches within one observation period rather than laying all eggs in just a single-mulch type (Figure 1).

Wild-caught beetles laid significantly more eggs on average when in small particle mulch rather than large particle and sand (Table 1). All 24 wild-caught females laid eggs when given a choice between substrates of different particle size and demonstrated a significant preference for small mulch substrate (Figure 1; χ^2^ = 61.00, df = 4, *p* < 0.0001). Only two beetles laid eggs in both mulch options within the same trial, with 20 of the remaining beetles ovipositing in small particle size mulch, 2 beetles in the large particle size mulch, and 0 beetles in sand.

On average, laboratory-reared females laid significantly more eggs in small particle mulch compared to large particle mulch and sand, also showing a preference for small particle substrate (Table 1). Of the 24 laboratory-reared beetles given a choice between small and large particle size substrate, 6 beetles did not lay eggs (Figure 1; χ^2^ = 21.00, df = 3, *p* = 0.0001). The remaining beetles chose one of the two mulch substrates, with the majority (*n* = 15) ovipositing in small particle size mulch and 3 ovipositing in large particle size. No beetles laid eggs in sand.

### 3.2. Biometrics

Wild-caught beetles laid more eggs on average (± SE), 43 ± 5, than laboratory-reared beetles, 8 ± 2 (F_1,46_ = 36.99, *p* < 0.0001). In terms of their biometrics, wild-caught beetles were different from laboratory-reared beetles (MANOVA: F_3,38_ = 13.97; *p* < 0.0001). Wild-caught beetles were larger than laboratory-reared beetles in terms of depth (F_1,40_ = 5.10, *p* = 0.0296), weight (F_1,40_ = 37.43, *p* < 0.0001) and length (F_1,40_ = 14.60, *p* = 0.0005) (Table 2). The two groups were not different with regard to width (F_1,40_ = 0.80, *p* = 0.3776) (Table 2). No suitable model using biometric parameters could be generated to predict fecundity for wild-caught (F_9,14_ = 2.23, *p* = 0.0832) or laboratory-reared beetles (F_5,12_ = 0.52, *p* = 0.8377).

## 4. Discussion

Understanding CRB oviposition behavior is essential, as it could aid in developing appropriate localization and management methods for the prevention and control of oviposition sites, and the reproduction and spread of CRB populations in Hawaiʻi. This study is the first to examine and demonstrate both laboratory-reared and wild-caught *O. rhinoceros* oviposition preference in relation to substrate, and reveals that CRB will discern between medium when given different particle sizes, which appears to be consistent with other studies of scarab behavior [8,14].

Wild-caught and laboratory-reared CRB both prefer substrate made up of small particle size rather than large, and actively avoid sand. Females could prefer a smaller particle to provide a suitable environment for larval development and pupation. This could also provide a more easily handled material to pack eggs with [7]. This preference suggests that piles of small particle material may be more likely as a CRB breeding site, and that managing particle size within mulch piles could be considered a factor for improved CRB management. For example, milling plant material to consistently large particle size may deter and reduce reproductive success of the beetles. There is a likelihood that CRB will be able to adapt to this changed resource, so a suite of integrated pest management techniques should be incorporated together to reduce strong selective pressure. However, further research should be undertaken particularly to understand the suite of particle sizes and substrates that CRB can and will oviposit in, as this will enable more informed recommendations to reduce pest populations.

This study also underscored differences, which are often significant, between laboratory-reared and wild-caught insects, and was the first to make this comparison with coconut rhinoceros beetle. It is common for insects in a laboratory colony to become physically different or even exhibit different behavior [12,13,14]. In a paper examining the effects of laboratory rearing on factors like behavior and colony dynamics of paper wasps, *Polistes fuscatus*, differences in the laboratory and field environment were thought to skew the biological significance of results in experiments [15]. This was observed in our study, as wild-caught beetles were found to have a higher fecundity and larger size than laboratory-reared beetles. The developmental environment varied greatly between laboratory-reared and wild-caught CRB. The immature and adult stages of laboratory-reared CRB did not have the same handling, diet, space, environmental factors and stressors, microbiome, and predators etc. as wild-caught beetles. These factors could contribute greatly to the differences affecting oviposition behaviors and possibly other behaviors not yet studied. In the present case, the choice experiments indicated that developmental environment might lead to not only physical differences, but also meaningful differences in reproductive behavior in terms of fecundity.

Overall, these findings suggest that CRB produced in a laboratory colony may not be appropriate indicators for CRB oviposition behavior in the natural environment, and any studies conducted with laboratory-reared beetles should be interpreted with caution. Future studies looking at behaviors of laboratory-reared and wild-caught CRB could include lengthier comparisons following the life cycle and oviposition behaviors of these two beetle types.

In conclusion, this is the first laboratory study to investigate the individual oviposition preference of CRB not only in wild-caught beetles, but in laboratory-reared beetles as well. This study demonstrates that female CRB do discriminate on the basis of some environmental parameters in terms of oviposition site, e.g., substrate particle size. It is also the first direct comparison of laboratory-reared and wild-caught CRB behavior and demonstrates a difference in physical size and oviposition behavior within the two groups. Further studies should investigate the drivers behind these choices and variation in behavior.

## Figures and Tables

**Table 1:** Mean (±SE) number of eggs laid and mean (±SE) percent of total eggs laid by CRB. Comparison of the numbers of eggs was conducted using a Wilcoxon non-parametric test.

Treatment	Laboratory-Reared (*n* = 24)		Wild-Caught (*n* = 24)	
	# of Eggs	% of Eggs	# of Eggs	% of Eggs
Small Particle	8 ± 2	62.5 ± 0.1	41 ± 6	91.0 ± 0.1
Large Particle	1 ± 0	12.5 ± 0.1	3 ± 2	9.0 ± 0.1
Sand	0	0	0	0
	**t = 3.27, d.f. = 23, *p* = 0.0014**		**t = 6.18, d.f. = 23, *p* < 0.0001**	

**Table 2:** Average (±SE) body biometrics for CRB.

Measurement	Wild-Caught (*n* = 24)	Laboratory-Reared (*n* = 18)
Length (mm)	43.6 ± 0.6	40.4 ± 0.5
Width (mm)	19.4 ± 0.3	19.6 ± 0.2
Depth (mm)	15.2 ± 0.4	14.7 ± 0.2
Weight (g)	5.6 ± 0.3	3.67 ± 0.2
